# Effects of Purkinje Fiber Conduction Block on Cardiac Pump Function: Computational Modeling Study

**DOI:** 10.3390/bioengineering13040464

**Published:** 2026-04-15

**Authors:** Sandra P. Hager, Vahid Ziaei-Rad, Jenny S. Choy, Mengjun Wang, Ghassan S. Kassab, Lik Chuan Lee

**Affiliations:** 1 Department of Mechanical Engineering, Michigan State University, East Lansing, MI 48824, USA; ziaeirad@msu.edu (V.Z.-R.); lclee@msu.edu (L.C.L.); 2 California Medical Innovations Institute, San Diego, CA 92121, USA; jschoy@calmi2.org (J.S.C.); mwang@calmi2.org (M.W.); gkassab@3dtholdings.com (G.S.K.)

**Keywords:** cardiac modeling, finite element method, excitation–contraction coupling, electrophysiology, Purkinje fiber network, left bundle branch block, electromechanics coupling

## Abstract

Cardiac and hemodynamic conditions such as myocardial infarct, cardiomyopathy, hypertension, and aortic valve disease can impair conduction within the Purkinje fiber network and compromise left ventricular (LV) pump function. We developed a computational framework that couples electrical propagation in a structurally organized Purkinje fiber network with LV electromechanics to analyze the impact of conduction abnormalities on cardiac performance. A *baseline* simulation reproduced physiological activation patterns and pump indices consistent with healthy human data. Conduction block was then introduced at different locations within the Purkinje fiber network. LV pump function was strongly dependent on block location: *left bundle branch block (LBBB)* produced the largest reduction in ejection fraction (EF) (59% to 46%) and peak pressure (119 to 97 mmHg), whereas *left anterior fascicle block* caused smaller functional changes. Across simulations, myocardial activation delay and systolic dyssynchrony index (SDI) exhibited a nonlinear relationship with EF and myocardial strain. A threshold behavior was identified at a simulated LV activation duration of approximately 240 ms and an SDI of 8.4%, beyond which EF and strain decreased by about 5% relative to baseline. These findings provide a mechanistic framework to investigate how Purkinje fiber network conduction abnormalities influence LV pump dysfunction.

## 1. Introduction

Cardiac contraction is initiated and coordinated by the Purkinje fiber network, a specialized fast conduction system that helps ensure the rapid depolarization of myocardial tissue across the heart. Located within the subendocardial layer of the ventricles, the fibers are electrically coupled to the ventricular myocardium via Purkinje–myocardial junctions (PMJs), where myocardial tissue adjacent to the PMJs is stimulated when the corresponding Purkinje terminal node exceeds a predefined activation threshold [1,2,3]. During the normal activation sequence, the electrical signal propagates through the His bundle and bifurcates into the left and right bundle branches, which conduct towards the Purkinje fiber networks associated with the respective ventricles. The high electrical conductivity of the Purkinje fibers results in rapid depolarization of the endocardium, which leads to the propagation of the electrical signal through the myocardial wall to the epicardium. Total ventricular activation time in a healthy human heart ranges between 50 and 80 [ms], with Purkinje network activation completing within approximately 20–25 [ms] [4,5].

Various heart diseases, such as dilated cardiomyopathy [6], ischemic heart disease [7], and hypertensive heart failure [8], can cause conduction abnormalities or blocks at different locations in the cardiac conduction pathway, with the most common types being left bundle branch block (LBBB) and right bundle branch block (RBBB) [9,10]. In complete LBBB, electrical propagation through the entire left bundle is impaired or delayed, resulting in a loss of mechanical synchrony between the right and left ventricular activations [11]. More localized conduction disturbances, such as left anterior fascicular block (LAFB) and left posterior fascicular block (LPFB) [12], produce partial activation delays in specific fascicles. These conduction abnormalities, which are typically characterized by a prolonged QRS–complex duration, lead to mechanical dyssynchrony that impairs cardiac pump function and can contribute to heart failure progression. Clinically, LBBB and related fascicular blocks are major predictors of reduced ejection fraction and poor response to cardiac resynchronization therapy (CRT) [13,14,15]. Quantifying the mechanical consequences of such conduction delays remains important for improving the mechanistic understanding of factors influencing patient selection and pacing strategies.

Computational modeling has become an important tool for understanding the effects of electrical conduction abnormalities and pathological conditions such as LBBB. Electromechanical simulations that incorporate a detailed representation of the Purkinje system are particularly valuable for exploring how regional conduction abnormalities influence global cardiac function [16]. Previous studies have explored various strategies for generating Purkinje networks in silico, ranging from deterministic rule–based algorithms and fractal tree structures to stochastic growth algorithms that mimic anatomical variability [3,17,18]. These modeling approaches enable the study of activation timing, electrical conductivity heterogeneity, and the spatial distribution of Purkinje–myocardial junctions (PMJs), which are presumed to affect the overall activation pattern. These studies, however, have not investigated how conduction abnormalities in the Purkinje fiber network affect cardiac pump function in a systematic mechanistic manner.

To address this issue, we developed a computational framework that integrates a structurally organized Purkinje fiber network with ventricular electromechanics to understand how different types of conduction abnormalities occurring within the network affect cardiac pump function. The generated Purkinje fiber network includes a deterministic trunk originating near the His bundle and transitioning into stochastically branching terminal fibers across the endocardial surface. This hybrid approach introduces anatomically motivated Purkinje network organization while preserving reproducibility and controlled variation of distal branching geometry. Using the computational framework, we first established a baseline simulation case that reproduced physiological activation patterns and timings as well as cardiac pump function values reported in the healthy human heart. Subsequently, we simulated conduction blocks occurring at different locations within the Purkinje fiber network to assess their impact on cardiac pump function. Based on these simulation results, we identified a nonlinear relationship and a threshold behavior in myocardial activation time and systolic dyssynchrony index (SDI) beyond which cardiac pump function begins to deteriorate.

## 2. Materials and Methods

**Figure 1:**
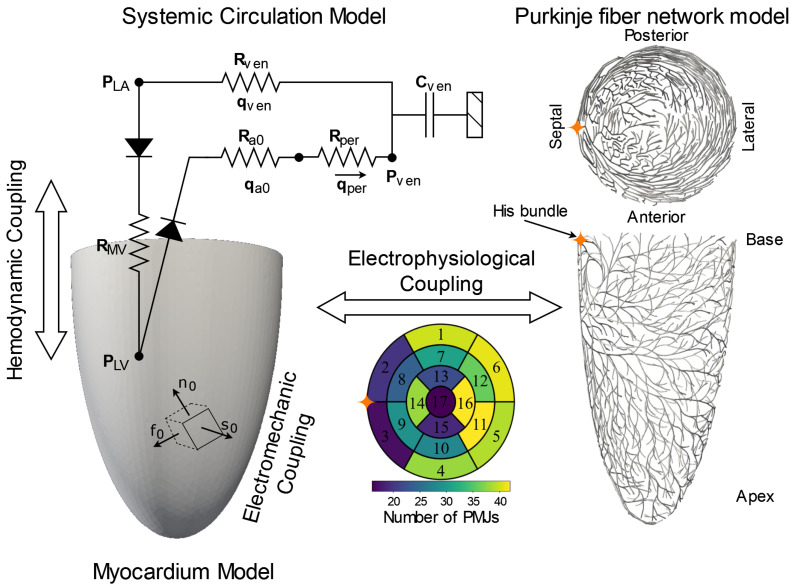
Schematic representation of the multiscale framework integrating three coupled physiological systems: a myocardium model, a Purkinje fiber network model, and a systemic circulation model. (**Left**): The myocardium model represents the electromechanical behavior of the left ventricle (LV), incorporating orthotropic fiber architecture defined by fiber ($f_{0}$), sheet ($s_{0}$), and sheet-normal ($n_{0}$) directions [20]. This model enables electromechanical coupling by linking electrical activation to active myocardial contraction. (**Right**): The Purkinje fiber network model represents the specialized conduction system originated at the His bundle and branching over the endocardial surface. (**Bottom center**): Excitation is transferred from the Purkinje system to the ventricular myocardium via Purkinje myocardial junctions (PMJs), as indicated in the bullseye map. (**Top center**): Systemic circulation model is represented as a lumped-parameter Windkessel model composed of resistance (R) and compliance (C), modeling pressure and flow dynamics through the arterial and venous compartments.

Figure 1 shows the computational framework consisting of three coupled physical systems, namely, the Purkinje fiber network generated based on the modification of a fractal tree algorithm [19], the left ventricle (LV), where the Purkinje fiber network is coupled at the endocardial surface via Purkinje–myocardium junctions (PMJs), and the systemic circulation into which the LV pumps blood and receives blood from. Electrical activation propagates from the Purkinje fiber network to the myocardium through the PMJs. Within the myocardium, the propagation of electrical waves leads to its mechanical contraction and the beating of the LV against the systemic circulation.

### 2.1. Geometry

#### 2.1.1. Purkinje Fiber Network

A Purkinje network on a ventricular surface was generated by modifying a fractal tree algorithm approach [19]. In the algorithm, geometrical parameters, namely, the branch length, branch angle and repulsion between mother and child branches are used to stochastically grow the Purkinje fiber network, which is then projected onto the endocardial surface. To reflect additional anatomical structures of the Purkinje network, based on experimental measurements, the algorithm was modified to enable a hybrid scheme. In this scheme, a deterministic trunk structure is first constructed, and the branches downstream are then generated stochastically using the original fractal tree algorithm. This deterministic trunk configuration reproduces the anatomically distinguishable left bundle branch (LBB), a dominant conduction structure in the septum [21]. In contrast to a pure stochastic fractal tree model, our modification enforces a physiologically motivated approximation compared to the prior stochastic branching model. The trunk originates from an a priori defined point on the LV endocardium near the base, approximating the anatomical location of the His bundle, and propagates downwards along the septum with a fixed length and orientation. This implementation provides greater control over the bifurcation point of the main trunk, where the Purkinje fiber network divides into the left anterior and posterior fascicles, while maintaining stochastic variation of the terminal branches. Terminal nodes of the generated network represent the PMJs that are distributed over the endocardial surface for the electrophysiological coupling of the two geometrical models. The hybrid deterministic–stochastic generation approach is consistent with experimental observations of subendocardial Purkinje network organization and current limitations in human anatomical reconstruction [21,22,23,24,25]. Consequently, many computational frameworks adopt fractal tree or hybrid generators that can be calibrated to morphometry and local activation timing, and only recently have patient–specific generation strategies begun to appear [18]. In this study, we therefore employ a hybrid deterministic–stochastic tree that preserves PMJ coverage and branching density consistent with experimental data [26] while remaining reproducible and computationally tractable. The bullseye plot in the center of Figure 1 shows the density of PMJs within the subendocardial layer subdivided into the 17 segments of the AHA regions, with segments 1–6 in the basal area, 7–12 in the mid-ventricular area, and 13–17 in the apex area. A total of [N≈582] PMJs were generated, corresponding to an average density of 7.46 [PMJs/cm^2^] across the LV endocardium, with locally higher densities in the mid–lateral and basal regions. This distribution matches physiologically consistent endocardial activation coverage across all AHA segments and avoids clustering of activation sites. The distribution is also consistent with quantitative experimental measurements of Purkinje–myocardial junction organization and density in large mammalian hearts [26]. All PMJs were located on the endocardial surface (depth ≈ 0 [mm]). The Purkinje fiber network was discretized using 3761 one-dimensional line elements with an average mesh size $h_{pj}$ = 1.1 [mm] (Figure 1, right). A larger mesh size was applied to the His bundle region prior to its bifurcation into the anterior and posterior fascicles, as well as in the lateral and apical regions. Table A1 in the Appendix A include a summary of the parameters used to generate the Purkinje fiber network.

#### 2.1.2. Left Ventricular Geometry

The LV geometry was idealized as a truncated ellipsoid similar to that in [27]. The myofiber field was generated using a rule-based Laplace–Dirichlet algorithm presented by [20] to produce a fiber vector field $f_{0}$ that has its angle (measured with respect to the circumferential direction) varying transmurally from −60 deg at the epicardium to 60 deg at the endocardium. Orthogonal to the fiber vector field $f_{0}$ lies the sheet vector field $s_{0}$ that defines the local myocardial sheet plane. The sheet-normal direction $n_{0}$ is defined as the cross product $f_{0}\times s_{0}$, completing the orthonormal basis at each quadrature point (Figure 1 left). The LV geometry was meshed with 33,528 tetrahedral elements for the electrophysiology model, with an average mesh size of $h_{ep}$ = 2.6 [mm]. The mechanics model was discretized using 13196 tetrahedral elements with an average mesh size of $h_{me}$ = 3.5 [mm].

### 2.2. Cardiac Electromechanics Governing Equations

#### 2.2.1. Cardiac Electrophysiology

Electrical activation and propagation in the myocardium and Purkinje fiber network were described using the monodomain formulation with the modified FitzHugh–Nagumo (FHN) model [28]. The governing equations, with indices pj denoting the Purkinje fiber network and myo denoting the myocardium, are given by:

(1) $$\frac{d\phi_{i}}{dt}\;=\nabla \cdot \left(D_{i}\;\nabla \;\phi_{i}\right)+f_{\phi }\left(\phi_{i},r_{i}\right)+I_{s_{i}},\;\;\;\mathrm{with}\;i\in \left\{pj,myo\right\}$$

(2) $$\frac{dr_{i}}{dt}=g_{r}\left(\phi_{i},r_{i}\right),\;\;\;\;\mathrm{with}\;i\in \left\{pj,myo\right\}$$

where $\phi_{i}$ represents the action potential, $r_{i}$ is the recovery variable of the individual cell, $D_{i}$ is the conductivity tensor in the individual domain, and $I_{s_{i}}$ is the stimulus current. To account for the anisotropic conduction in the myocardium, the conductivity tensor is given by:

(3) $$D_{myo}=d_{iso}\;I+d_{ani}f_{0}⊗f_{0}+\frac{d_{ani}}{\nu }s_{0}⊗s_{0}+\frac{d_{ani}}{\nu }n_{0}⊗n_{0},$$

where $D_{myo}$ represents anisotropic conductivity aligned with myocardial fiber architecture. This formulation is consistent with previous electromechanical models [29,30,31], while $d_{ani}$ is directly related to the microstructure, with parameter ν controlling the dampening along the sheet and sheet-normal directions. The coupling between the Purkinje fiber network system and myocardium occurs through the PMJs [32]. The myocardium is locally activated at each PMJ when the corresponding Purkinje terminal node is activated, i.e., when $\phi_{pj}>0.9$. A simulus current $I_{s_{myo}}$ is then applied to the surrounding myocardial tissue, namely:

(4) $$I_{s_{myo}}\left(x,t\right)=\sum_{j=1}^{N}\omega_{j}\left(t\right)\cdot I_{stim}\left(x\right),\;\mathrm{where}\left\{\begin{array}{cc} \omega_{j}\left(t\right)=1 & \mathrm{if}\;\;\phi_{pj}>0.9,\\ 0 & \mathrm{otherwise}. \end{array}\right.$$

Here, $\omega_{j}\left(t\right)$ represents a weighted scalar function that switches between an active state $\left(\omega_{j}\left(t\right)=1\right)$ or a state without stimulation of the myocardium (i.e., $\omega_{j}\left(t\right)=0\;\Rightarrow I_{s_{myo}}=0$). This formulation ensures physiologically consistent endocardial activation by explicitly representing discrete Purkinje–myocardial coupling sites, thereby avoiding purely diffusion-driven myocardial activation.

#### 2.2.2. Cardiac Mechanics

The mechanical behavior of the myocardium was modeled based on an active stress formulation where the second Piola–Kirchhoff (PK2) stress tensor S was additively split into the active ($S_{a}$) and passive ($S_{p}$) components, i.e.:

(5) $$S=S_{p}+S_{a}.$$

The passive component of S was described by:

(6) $$S_{p}=\frac{\delta \hat{W}\left(E_{e},p\right)}{\delta E_{e}},$$

with $\hat{W}$ as the strain energy density function with respect to the Green–Lagrange strain tensor $E_{e}$. The passive mechanical behavior was modeled using a phenomenological transversely isotropic Fung–type strain energy density function [33], namely:

(7) $$W\left(E_{e}\right)=\frac{c}{2}\left(\exp\left(Q\right)-1\right),\;\;Q=b_{f}E_{ff}^{2}+2b_{fs}\left(E_{fs}^{2}+E_{fn}^{2}\right)+b_{n}\left(E_{ss}^{2}+E_{nn}^{2}+2E_{sn}^{2}\right),$$

where $c,b_{f},b_{fs}$, and $b_{n}$ are the myocardial parameters, and $E_{ff},E_{fs},E_{fn},E_{ss},E_{nn}$, and $E_{sn}$ are components of the Green–Lagrange strain tensor in each material direction. Local incompressibility constraint on the myocardial tissue was enforced using a Lagrange multiplier approach such as:

(8) $$\hat{W}\left(E_{e},p\right)=W\left(E_{e}\right)-p\left(J-1\right).$$

J=det(F) is the determinant of the deformation gradient $F\left(X,t\right)=\nabla_{X}u\left(X,t\right)+I$, with the displacement field $u:\Omega_{0}\times \left[0,T\right]\to R^{3}$, defined over time and with respect to the material points in the reference configuration $X\in \Omega_{0}$, where $\Omega_{0}\subset R^{3}$. To model the active mechanical behavior of the myocardium, we used a phenomenological model [27,34] in which the active second PK stress tensor is given by:

(9) $$S_{a}=T\left(t,t_{init}\left(X\right),C_{a0},E_{ff},T_{max}\right)f_{0}⊗f_{0}\;,$$

where

(10) $$T\left(t,t_{init},C_{a0},E_{ff},T_{max}\right)=T_{max}\frac{C_{a0}^{2}}{1+EC_{a50}^{2}\left(E_{ff}\right)}\frac{1-cos\left(\omega \left(t,t_{init},E_{ff}\right)\right)}{2}.$$

In the above equation, $T_{max}$ is the isometric tension at maximum sarcomere length, $C_{a0}$ is the peak intracellular calcium concentration, and $\omega \left(t,t_{init},E_{ff}\right)$ describes the time-dependent activation phase as:

(11) $$\omega =\left\{\begin{array}{cc} \pi \frac{t}{t_{0}} & \mathrm{when}\;\;0\leq t<t_{0},\\ \pi \frac{t-t_{0}-t_{r}}{t_{r}} & \mathrm{when}\;\;t_{0}\leq t<t_{0}+t_{r},\\ 0 & \mathrm{when}\;t_{0}+t_{r}\leq t, \end{array}\right.$$

where $t_{0}$ is the time–to–peak tension and $t_{r}=ml+b$ is the duration of relaxation, with m,b defined as constants and *l* defined as current sarcomere length. The length-dependent calcium sensitivity of the myocardium is given by:

(12) $$EC_{a50}\;=\frac{{\left(C_{a0}\right)}_{max}}{\sqrt{\exp\left(B\left(l-l_{0}\right)\right)-1}}\;,$$

where $l_{0}$ is the reference sarcomere length, and *B* is the sarcomere length sensitivity coefficient. Similar to [30], coupling between electrophysiology and mechanics was implemented based on the local activation time $t_{init}\left(X\right)$ that starts when $\phi_{myo}\left(X,t\right)>0.9$.

### 2.3. Numerical Implementation

The coupled electromechanical problem was implemented in FEniCS [35], an open–source finite element solver library, using an implicit time integration scheme for the electrophysiological model. The resulting nonlinear system was solved monolithically using a Newton–Raphson method. Mechanical equilibrium was enforced using a mixed finite element formulation with a displacement-pressure formulation to account for nearly incompressible myocardial tissue properties. A Robin–type boundary condition was applied on the epicardial surface to represent pericardial constraint, while left ventricular pressure was prescribed on the endocardial surface. Additional details of the numerical formulation, including the weak forms, stabilization, and boundary condition implementation, are provided in Appendix A.

### 2.4. Closed-Loop Circulatory Model

To simulate physiologically realistic ventricular loading conditions, the LV was coupled with a closed–loop lumped–parameter circulatory model using $P_{LV}$ and the LV cavity volume. Details of the formulation for the lumped–parameter circulatory model can be found in [27]. To ensure comparability across all conduction scenarios, the total stressed blood volume and venous filling pressure were kept constant across all simulations.

### 2.5. Simulation Cases

**Figure 2:**
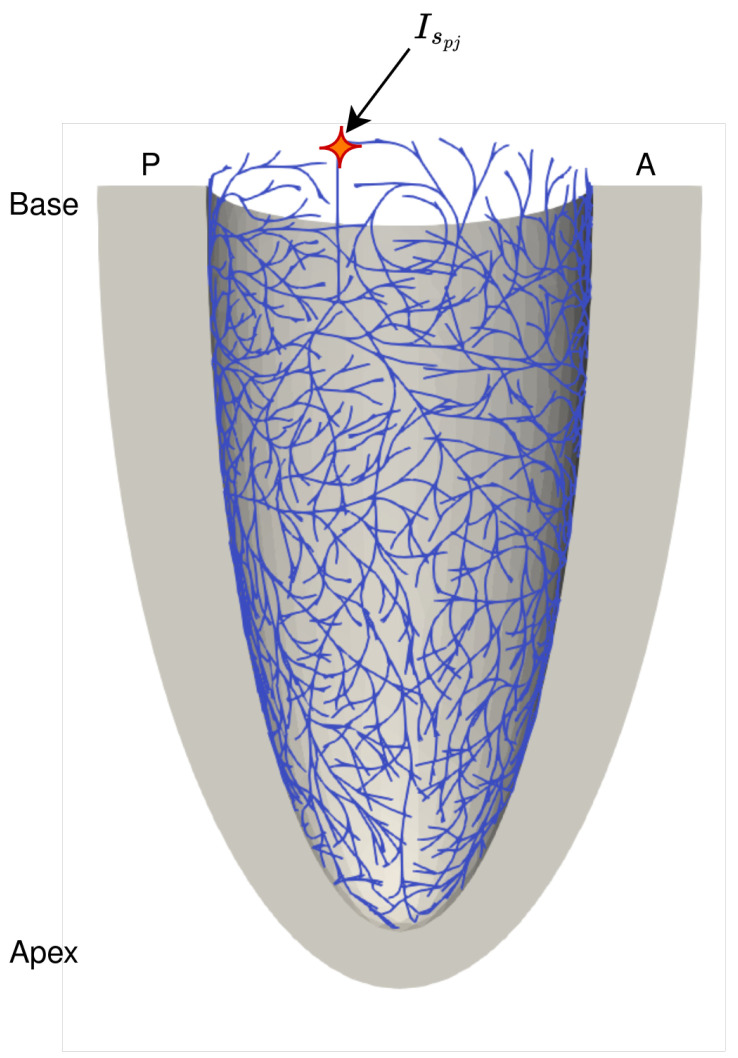
Schematic of Purkinje fiber network embedded on the endocardial surface of the left ventricle in posterior (P)–anterior (A) view with the His bundle highlighted (orange star). Electrical stimulation is initiated at the base of the trunk (orange star with red outline), corresponding to the application of an input current $I_{s_{pj}}$, triggering activation propagation throughout the network.

A baseline simulation model was first established to benchmark its prediction against experimental measurements. Thereafter, simulations with different degrees of complete blocks were performed. For all simulations, the Purkinje fiber network was electrically stimulated ($I_{s_{pj}}$) at the His bundle region (Figure 2).

#### 2.5.1. Baseline Simulation Case

In the baseline simulation case, the conductivity tensor associated with the myocardium was calibrated to replicate the activation pattern found in experimental measurements in [4], which provides detailed isochronal activation maps of the human heart. The conductivity tensor associated with the Purkinje fiber network was calibrated to match the activation time under normal sinus rhythm found in [5]. The simulation parameters are summarized in Appendix A, Table A2. In addition to the conduction parameter of the Purkinje fiber network and the myocardium, the passive and active mechanical properties of the myocardium were calibrated to replicate the mechanical pump function of a healthy heart, indexed by ventricular pressure, cavity volume, ejection fraction and stroke volume [36].

#### 2.5.2. Conduction Block Simulation Cases

Simulation cases were performed to assess the impact of different locations of conduction block in the left bundle branch (LBB) on LV electromechanics. Conduction parameters are summarized in Appendix A, Table A3.

**Figure 3:**
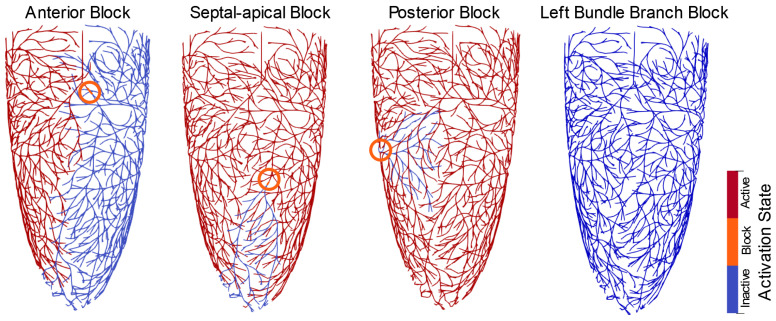
Simulation of pathological variations in Purkinje fiber network conduction, including regional left bundle branch blocks: anterior (*LAFB*), septal-apical (*LSAFB*), posterior (*LPFB*), and total block (*LBBB*). The orange circles indicate the locations of localized conduction block within the Purkinje fiber network. Red and blue lines represent active and inactive regions, respectively. Each case disrupts the normal activation sequence and illustrates how localized Purkinje fiber network deficits impair coordinated ventricular depolarization.

Conduction block was simulated by setting the local electrical conductivity of specific branches to zero. Four locations of blockage were simulated (Figure 3), namely:

- Anterior block (*LAFB*), where conduction was disrupted in Purkinje fibers located at the anterior wall, delaying activation across anterior and anterolateral segments.
- Septal–apical block (*LSAFB*), where conduction was disrupted in Purkinje branches terminating near the apical septum, affecting early apical activation and disturbing basal–to–apical timing.
- Posterior block (*LPFB*), where conduction was disrupted in Purkinje fibers innervating the posterior and inferolateral walls, thereby affecting mid–to–basal activation in posterior regions.
- Total block (*LBBB*), where the entire left bundle was blocked, to simulate a complete failure of rapid subendocardial activation.

#### 2.5.3. Sensitivity Analysis of Purkinje Fiber Network Geometry

For evaluation of robustness of the proposed framework with respect to variations in Purkinje fiber network structure, a sensitivity analysis was performed using 100 independently generated Purkinje fiber networks. The key parameters that were investigated included *Repulsion* (*r*), *Length* (len) and *Generation order* (*N*) in the mid–section of the Purkinje fiber network, an area that is associated with the overall geometry of the network. After generation of the individual networks, we performed an electrophysiological simulation in all cases using identical parameter settings as in the *baseline* case to isolate the effect of Purkinje fiber network structure.

## 3. Results

### 3.1. Baseline Simulation Case

#### 3.1.1. Electrophysiological Activation Pattern

**Figure 4:**
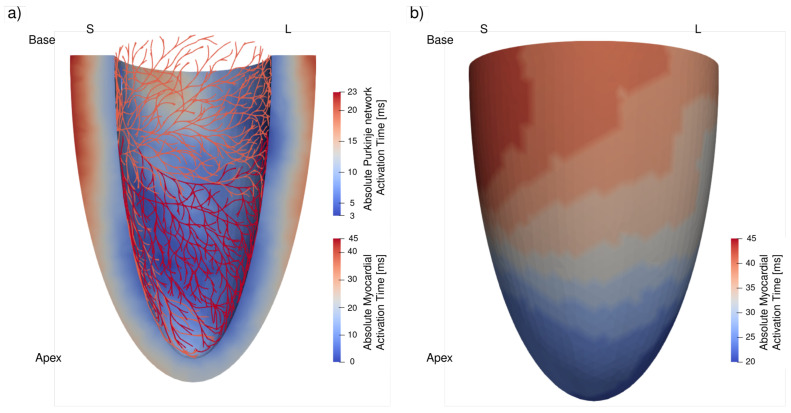
Baseline ventricular activation map showing transmural and apicobasal propagation patterns in a septal (S)–lateral (L) view. The activation times were computed using conduction velocities calibrated to reproduce the physiologically observed range of activation delays. (**a**): Activation map of Purkinje fiber network and transmural activation map of myocardium. (**b**): Epicardial activation map. All times are given in milliseconds.

Figure 4 shows the predicted activation time maps of the Purkinje fiber network and the LV. The Purkinje fiber network was completely activated after 23 [ms], and activation time was relatively homogeneous. This behavior can be associated with a median conduction velocity of 3.2 [m/s], comparable to the reported range of 2–4 [m/s] [37,38]. On the other hand, 90% of the LV activated after 40 [ms] with respect to the first breakthrough occurring at the PMJ. The median computed conduction velocity of 0.4 [m/s], approximately tenfold lower compared to velocities across the Purkinje fiber network, is comparable with literature values reported in healthy human myocardium [4,39]. In the LV, activation time increased transmurally from the endocardium to the epicardium, with the transmural gradient most prominently in the basal region. At the epicardium, the apical region had the smallest activation time, whereas the septal–base region had the largest activation time.

#### 3.1.2. Cardiac Mechanics

**Figure 5:**
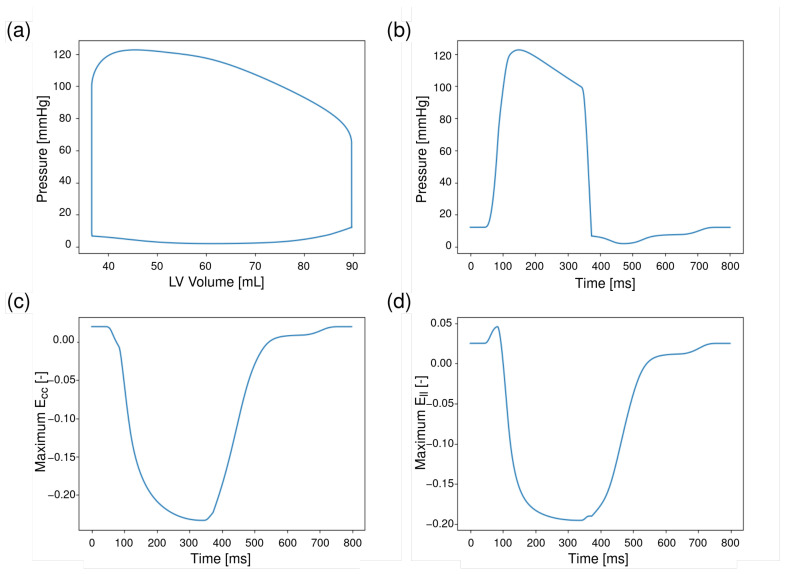
(**a**,**b**) Simulation of left ventricular pressure–volume (PV) loops (**a**) and pressure traces (**b**) for baseline case over a cardiac cycle. Simulated PV loop shows the relationship between left ventricular (LV) pressure and volume for a steady–state cardiac cycle. Corresponding LV pressure curve over time for the same cardiac cycle. (**c**,**d**): Peak (maximum shortening; most negative) LV strains in the baseline case over a cardiac cycle. (**c**): Maximum circumferential strain ($E_{cc}$). (**d**): Maximum longitudinal strain ($E_{ll}$) in LV. Times are given in milliseconds, pressures are in millimeters of mercury, and LV cavity volume is in milliliters.

Figure 5a,b show the results for the LV pressure–volume (PV) loops and LV pressure traces at a time–periodic steady state. The LV peak systolic pressure is 123 [mmHg], end–systolic volume is 37 [mL], and end–diastolic volume is 90 [mL], which resulted in a stroke volume of 53 [mL] and ejection fraction (EF) of 59%.

The simulated LV end-diastolic pressure (LVEDP) was 12 [mmHg], which lies within the normal physiological range for a healthy ventricle (4–12 [mmHg] [40]). Figure 5c,d shows the global strain waveforms in the circumferential ($E_{cc}$, (c)) and longitudinal ($E_{ll}$, (d)) directions in the baseline case. The maximum shortening of the LV, with an absolute peak $E_{cc}$ of 0.23 and an absolute peak $E_{ll}$ of 0.19, occurred at 332 [ms] after end–diastole.

### 3.2. LBBB Simulation Cases

#### 3.2.1. Electrophysiological Activation Pattern

**Figure 6:**
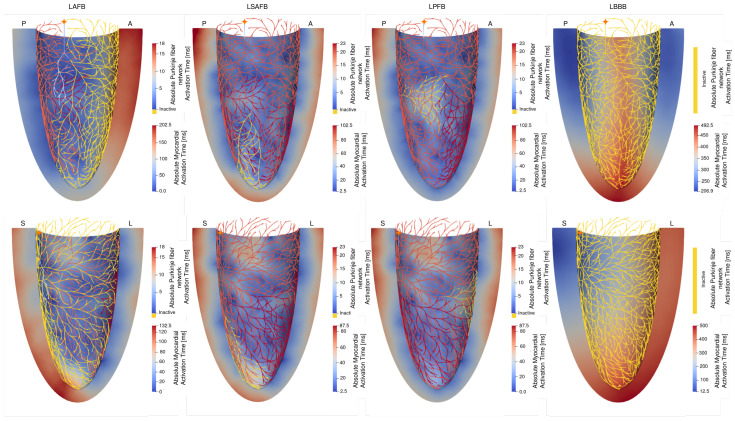
Activation maps illustrating the electrophysiological consequences of bundle branch block (BBB) under different pathological configurations. In the Purkinje fiber network, yellow regions indicate electrically inactive regions. The simulations include *LAFB*, *LSAFB*, *LPFB*, and total Purkinje fiber network block (*LBBB*); color maps show activation times in both the Purkinje fibers and the myocardium, with blue indicating early and red late activation. (**Top**): Posterior (P)–anterior (A) view. (**Bottom**): Septal (S)–lateral (L) view. The orange star highlights the position of the His bundle.

Figure 6 shows the activation time maps for the different conduction blocks in the Purkinje fiber network, with inactive regions highlighted in yellow. In the *LAFB* case, the posterior branches of the Purkinje fiber network were fully activated after 18 [ms], with a median conduction velocity of 3.9 [m/s], which remains within the range of reported healthy Purkinje fiber networks. However, the faster activation in this case might originate in the structure of the activated fascicles of the network. As a result of the various fascicle blocks, there was a substantial delay in myocardial activation, where 90% of the myocardium was activated only after 170 [ms]. The activation pattern of LV also differs significantly from that in the baseline case, where the anterior endocardium was activated later in the cycle due to the conduction block.

In the *LSAFB* case, the Purkinje fiber network (excluding the blocked region) was activated after 23 [ms], and 90% of the myocardium was activated after 70 [ms]. Due to the septal–apical location of the blocked fascicle, activation in the apical region was delayed compared to the baseline. In the *LPFB* case, the Purkinje fiber network (except for the blocked region) was activated after 23 [ms], and 90% of the myocardium was activated after 70 [ms] with respect to the first breakthrough occurring at the PMJ. In *LSAFB* and *LPFB*, we did not observe changes in the median conduction velocity of the Purkinje fiber network compared to the *baseline* case (3.2 [m/s]). The LV activation pattern was not very much affected in this case. In the *LBBB* case, the entire Purkinje fiber network remained inactive, resulting in the absence of conduction within the network. In this case, external stimulation was applied to the epicardium in the septal region. Furthermore, 90% of the myocardium was activated after 420 [ms], where there was substantial delay in activation in the free wall region (located furthest from the earliest activated septal region). The median local conduction velocity of the myocardium was 0.2 [m/s] for each of the Purkinje fiber blocks. These values are comparable to velocities within ischemic ventricles [41,42,43,44].

#### 3.2.2. Cardiac Mechanics

**Figure 7:**
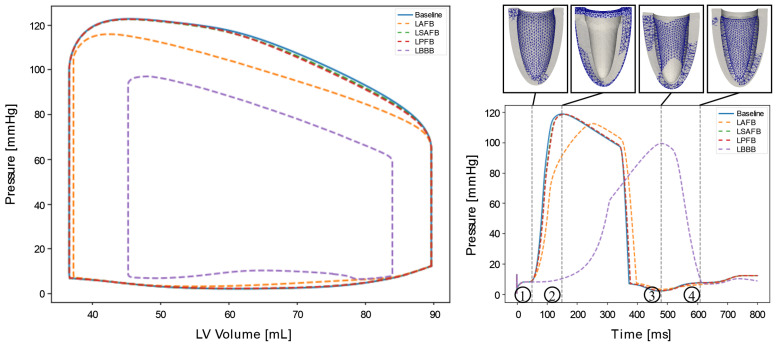
(**Left**): Pressure–volume (PV) loops of the left ventricle for *baseline* (blue solid line), *LAFB* (orange dashed line), *LSAFB* (green dashed line), *LPFB* (red dashed line), and *LBBB* (purple dashed line) case. (**Right top**): Comparison of ventricular displacement fields between baseline activation (gray geometry) and total Purkinje fiber block (blue wireframe) at four time points throughout the steady-state cardiac cycle (t = 50 [ms], 149 [ms], 479 [ms], and 610 [ms]). Displacement fields are shown from a septal–lateral view. (**Right bottom**): Corresponding left ventricular pressure waveforms over a steady–state cardiac cycle with the *baseline*, *LAFB*, *LSAFB*, *LPFB*, and *LBBB* simulations marked as solid blue, dashed orange, dashed green, dashed red, and dashed purple, respectively. The numbered markers (1–4) denote key phases of the cardiac cycle: (1) end–diastole: *baseline* and *LAFB*, (2) peak systole: *baseline*, (3) peak systole: *LBBB*, and (4) end–isovolumetric relaxation: *LBBB* case.

Figure 7 shows a comparison of left ventricular pressure–volume (PV) loops (left) and corresponding pressure waveforms (right) between the *baseline*, *LAFB*, *LSAFB*, *LPFB* and *LBBB* cases. Compared to the baseline case, EF in the *LAFB* case was slightly lower (58% vs. 59%), while in the *LBBB* case, it was substantially lower (46% vs. 59%). For the *LSAFB* case and *LPFB* case, the EF compared to the *baseline* case did not show any changes (59% vs. 59%). In the *LAFB* case, the PV loop shows that pressure during most of the ejection phase was lower compared to the baseline case, whereas in the *LBBB* case, pressure was substantially lower. The isovolumic contraction and relaxation phases in the *LBBB* case also occurred at lower and higher LV volumes, respectively. A comparison of the pressure waveforms shows that the rate of increase in pressure and peak pressure was reduced in both the *LAFB* and *LBBB* case (*LAFB*: 252 [ms] and 112 [mmHg] and *LBBB*: 479 [ms] and 97 [mmHg]).

To assess the relative contribution of Purkinje versus myocardial conduction changes, we performed an additional simulation in which LBBB was modeled solely by suppressing Purkinje conduction without modifying the myocardial diffusion tensor. In this case, only a minor reduction in ejection fraction was observed compared to baseline. This indicates that disruption of rapid endocardial activation alone is insufficient to reproduce the degree of mechanical impairment typically associated with LBBB, as myocardial conduction can still support slower activation propagation. This highlights the importance of impaired myocardial activation in affected regions.

Figure 7, top right, compares LV deformation between the baseline (gray) and *LBBB* cases across the cardiac cycle. During early isovolumetric contraction, the baseline LV begins to contract, while the LBBB LV is still passively filling. At peak pressure, the baseline LV shows reduced length and increased wall thickness, whereas the *LBBB* LV just begins contraction. At early filling, the *LBBB* LV contracts asymmetrically, with a thicker septum. Midway through filling in the *baseline* case, the *LBBB* LV enters diastole and begins to fill.

**Figure 8:**
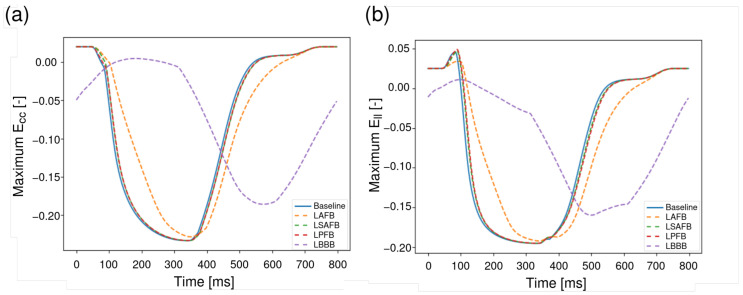
Comparison of maximum strains in the LV between *baseline* (blue line), *LAFB* (dotted orange line), *LSAFB* (dotted green line), *LPFB* (dotted red line), and *LBBB* (dotted purple line) conditions over two cardiac cycles. (**a**): Maximum circumferential strain ($E_{cc}$). (**b**): Maximum longitudinal strain ($E_{ll}$). The maximum stain corresponds to maximum LV shortening (most negative strain). Time is shown in milliseconds.

Figure 8 shows a comparison of global peak strain waveforms in the circumferential ($E_{cc}$, (**a**)) and longitudinal ($E_{ll}$, (**b**)) directions between the *baseline*, *LAFB*, *LSAFB*, *LPFB*, and *LBBB* cases. In the *baseline* case, the peak absolute values corresponding to maximum LV shortening (most negative strain) were $E_{cc}=0.23$ and $E_{ll}=0.19$, with a time to peak of 332 [ms] relative to end–diastole. Only the *LBBB* case exhibited a substantial reduction in these maximum shortening values ($E_{cc}$: 0.19 and $E_{ll}$: 0.16) and a substantial increase in the time to peak (500 [ms]). For the other cases, the absolute peak values and the time to peak value did not change much, and only the *LAFB* case showed a reduction in the strain rate.

**Figure 9:**
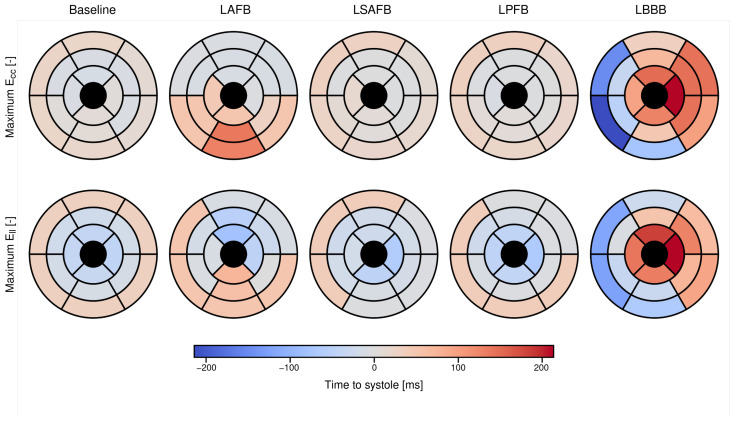
AHA 17–segment polar plots showing the regional time to peak systole across different conduction scenarios. The top row presents the timing maps for circumferential strain ($E_{cc}$), while the bottom row shows longitudinal strain ($E_{ll}$). Each column corresponds to a different condition: *Baseline*, *LAFB*, *LSAFB*, *LPFB* and *LBBB*. Color bars indicate the time to peak systole in milliseconds relative to global end–systole, with blue indicating early activation and red being associated with peak strain after reaching end–systole.

Figure 9 shows regional distributions of time delay (i.e., difference between time to peak strain $t_{peak\;strain,i}$ for each segment *i* and end-systolic time $t_{systole}$) for both the circumferential ($E_{cc}$) and longitudinal ($E_{ll}$) strains for the simulation cases. Compared to the *baseline* case, which shows a relatively small and uniform distribution of time delay across all segments in both $E_{cc}$ and $E_{ll}$, the *LAFB* and *LBBB* cases exhibit increased delays and greater variability across segments. The *LBBB* case shows the largest dispersion in time delay across all segments, ranging between −200 to 200 [ms]. The SDI of the *LBBB* case is the largest (14.5%), followed by the *LAFB* (5.87%), whereas the SDI values for the *LPFB* (1.65%) and *LSAFB* (1.42%) cases are close to that found in the *baseline* case (1.51%).

Table 1 shows the systolic dyssynchrony index (SDI), a common index for mechanical dyssynchrony, computed for the simulation cases based on $E_{cc}$ and $E_{ll}$. The SDI is calculated as the standard deviation of the time delay across all segments [45], i.e.,

(13) $$SDI_{E_{cc},norm}=\frac{\sigma \left(t_{peak\;strain,i}-t_{systole}\right)}{t_{RR}}\times 100.$$

The SDI of the *LBBB* case is the largest (14.5%), followed by the *LAFB* (5.87%), whereas the SDI values for the *LPFB* (1.65%) and *LSAFB* (1.42%) cases are close to that found in the *baseline* case (1.51%).

### 3.3. Relationship Between Dyssynchrony and Global Cardiac Function

**Figure 10:**
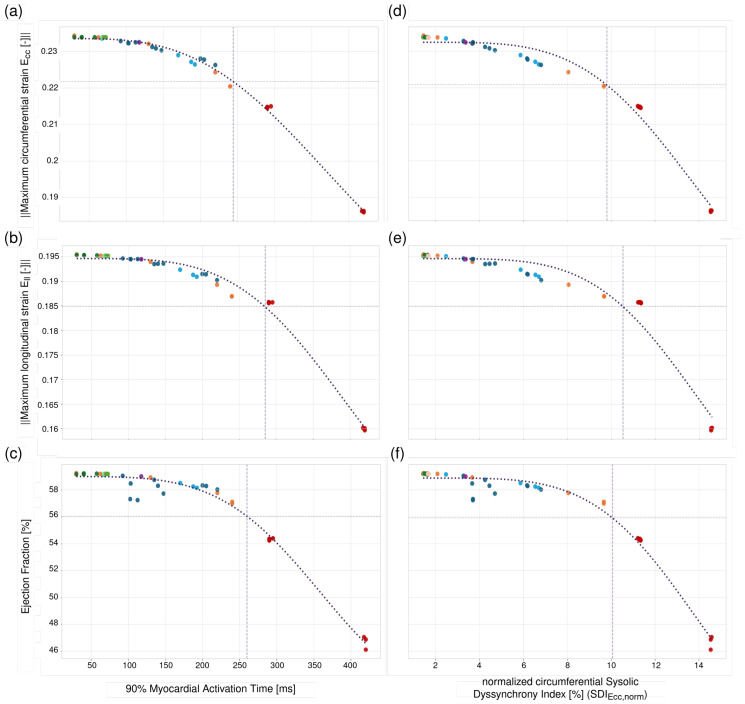
Sensitivity analysis of left ventricular activation times in relation to mechanical parameters. Maximum circumferential strain ($E_{cc}$, (**a**)) and maximum longitudinal strain ($E_{ll}$, (**b**)) plotted over myocardial activation times. Ejection fraction (**c**) changes visualized across a range of myocardial activation times. Right: Sensitivity analysis of left ventricular systolic dyssynchrony index with respect to the circumferential direction ($SDI_{E_{cc},norm}$) in relation to mechanical parameters. Maximum circumferential strain ($E_{cc}$, (**d**)) and maximum longitudinal strain ($E_{ll}$, (**e**)) plotted over $SDI_{E_{cc},norm}$. Ejection fraction (**f**) changes visualized across a range of $SDI_{E_{cc},norm}$. The purple line in all the plots shows the fitted trend behavior of the individual parameter with respect to either, with increasing myocardial activation time or $SDI_{E_{cc},norm}$ in [%] and the crosshair marking the point of 5% decrease from the baseline. All times in the plots are given in milliseconds. Colors represent different parameter configurations used in the simulations. Not all parameter variations are uniquely distinguished by color, and some cases may share same color despite differences in individual parameters (see Appendix A, Table A6.

Figure 10a–c shows an inverse relationship between indices of cardiac function, namely, $E_{cc}$, $E_{ll}$, and EF, with myocardial activation time (i.e., time taken for 90% of the myocardium to be activated) and SDI (Figure 10d–f for different types of conduction blocks. For $E_{cc}$ and EF, there was a 5% decline in its values when the activation time was greater than 240 [ms]. This time point marks the transition point of the trend line. To quantify this drop, the relative percentage deviation from baseline (early activation, <100 [ms]) was defined as:

(14) $$\Delta_{M}\left(90\%\;AT\right)\;=\;\frac{M_{ref}-M\left(90\%\;AT\right)}{M_{ref}}\;\times 100\%,\;M\;\in \left\{EF,{|E}_{cc}\left|,\right|E_{ll}|\right\}.$$

Here, $M_{ref}$ corresponds to the value at *baseline* (e.g., $EF_{ref}=0.592$). Beyond 240 [ms], a decline in EF and $E_{cc}$ by more than 5% was found, whereas a decline in $E_{ll}$ by over 5% was found beyond 285 [ms] (see Appendix A, Table A4). The relationships of the cardiac function indices and SDI are similar to those found with myocardial activation time, where there is a 5% decline in $E_{cc}$, $E_{ll}$, and EF once SDI exceeds 8.4% (see Appendix A, Table A5).

### 3.4. Quantitative Summary of Cardiac Function and Comparison with Experimental Data

For a consolidated overview of the simulation results with respect to the cardiac function and mechanical indices, we summarized and compared our results with reported data from the literature (Table 2). The *baseline* case reproduced key metrics within physiological ranges for ejection fraction, ventricular volumes, peak pressures, and systolic dyssynchrony (SDI) index related to circumferential strain, demonstrating consistency with experimental and clinical data. Conduction block scenarios exhibited reduced pump function and increased SDI, consistent with reported pathological trends. Notably, the *LBBB* case reproduced both reduced ejection fraction and elevated dyssynchrony levels comparable to those observed in heart failure patients, indicating that the model captures key features of pathological ventricular dysfunction. These results demonstrate that the framework reproduces physiologically and clinically consistent trends in ventricular function and provides a robust framework for mechanistic investigation of conduction abnormalities, such as *LBBB*.

### 3.5. Sensitivity Analysis of Purkinje Fiber Network Geometry

**Figure 11:**
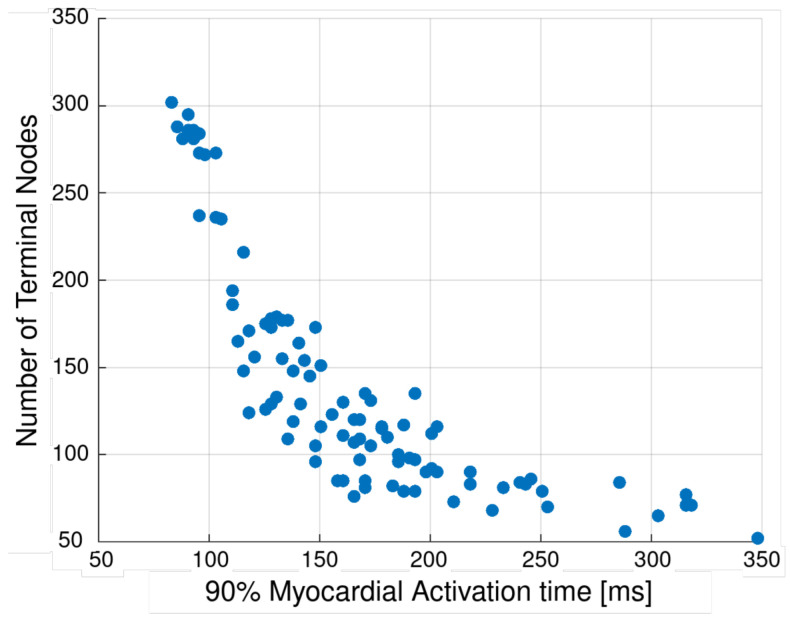
Relationship between the number of terminal nodes (Purkinje myocardium junctions) and myocardial activation time across 100 generated Purkinje fiber networks. Increased terminal node density is associated with reduced activation time, indicating that network density is a primary driver for activation dynamics.

A strong relationship was observed between the number of terminal nodes (representing PMJs) and 90% myocardial activation time (Figure 11), with increased terminal node density associated with reduced activation time (≈−0.78).

In contrast, other network parameters influence myocardial activation indirectly through their effect on the resulting terminal node density. Detailed parameter combinations and corresponding activation metrics are provided in Appendix A, Table A7, with statistical relationships summarized in Appendix A, Table A8. These results indicate that variations in Purkinje fiber network structure primarily influence the timing of myocardial activation. In combination with the previously established relationship between activation time, systolic dyssynchrony index (SDI), and cardiac pump function, this supports the idea that the overall trends in ventricular function are preserved across different network realizations.

## 4. Discussion

We developed a computational framework coupling left ventricular electromechanics with the propagation of electrical waves in the Purkinje fiber network, enabling detailed analysis of conduction abnormalities and consequently the impact on the cardiac pump function. Using the framework, we found that cardiac pump function declines with increasing myocardial activation time and the systolic dyssynchrony index (SDI), where the rate of deterioration becomes larger than 5% at thresholds greater than 240 [ms] and 8.4%, respectively. The extent of cardiac pump function deterioration at longer activation times depends strongly on the location of the conduction block, with blocks near the His bundle producing the most severe reduction of cardiac output. These results establish a mechanistic link between specific Purkinje conduction abnormalities and quantifiable losses in cardiac pump function.

### 4.1. Comparison of Activation Patterns with Measurements from Experiments and Clinics

The *baseline* simulation is able to reproduce the key features of Purkinje and ventricular activation pattern found in the experiment benchmark (Figure 4). Specifically, the predicted activation time of the Purkinje fiber network (23 [ms]) agrees with optical mapping data reported by [5], where the average activation time in healthy hearts was measured as 20.1 ± 7.0 [ms]. In the myocardium, the observed activation time of approximately 40 [ms] (for 90% of the LV) and the total myocardial activation time of 52.5 [ms] agrees with measurements on isolated human hearts (55 [ms] for the total myocardial activation) [4]. Model prediction of the apicobasal and transmural variation of activation time is also in agreement with experimental studies showing similar patterns [51,52].

Localized blocks within the Purkinje fiber network produce distinct activation patterns in both the Purkinje network and ventricular myocardium (Figure 6). Besides the *LBBB* case, where the entire Purkinje fiber network was not activated, the *LAFB* case showed the most pronounced increase in myocardial activation time, with delays up to around 185 [ms]. This delay occurs because the electrical wave could only propagate from the posterior to the anterior side through the myocardial wall in the LV, which is slower (than those directly from the Purkinje fiber network). This behavior is consistent with experimental studies reporting mechanical dyssynchrony and impaired hemodynamics even in partial fascicular blocks [9,31]. In contrast, *LSAFB* and *LPFB* cases show little impact on global activation times, as the blocks occur further downstream and the affected regions in those cases are small in comparison. This result is consistent with high-resolution electro-anatomical mapping studies of conduction blocks in the posterior fascicles, which produce subtle alterations in the activation pattern [53]. The most severe alterations were found in the *LBBB* case, where the activation of the LV relied entirely on the propagation of the electrical wave in the myocardium. These results are consistent with observed depolarization patterns in patients with *LBBB* in other computational studies, where electrical dyssynchrony resuleds in a delayed excitation of the LV free wall and reduced contractile coordination [54].

### 4.2. Impact of Conduction Block on Cardiac Pump Function

The simulation results also show that conduction blocks in the Purkinje fiber network affect cardiac function depending on the location. In the baseline case with normal activation, myocardial contraction is synchronous and symmetrical, which translates to a rapid rise in pressure during isovolumic contraction. The resultant EF (59%), myocardial strains ($E_{cc}$: 0.23 and $E_{ll}$: 0.19) and SDI ($SDI_{E_{cc},norm}$: 1.51%) are all comparable with measurements for a healthy human [48,55,56,57]. In the *LBBB* case, a significant intraventricular mechanical dyssynchrony during a cardiac cycle is observed with an SDI of 14.5%, which is well above the 6% threshold used to define dyssynchrony in clinics [48]. The delayed electrical activation of the LV results in asynchronous contraction across the myocardial wall, which leads to substantial changes in the PV–loop morphology. The pressure waveform also becomes more blunted, with lower peak values compared to the baseline case. The difference in ejection fraction and peak pressure observed across conduction block cases reflects the alteration of electrical activation rather than changes in filling conditions. All simulations were performed under identical preload and boundary parameters, ensuring that mechanical variations arise solely from the altered electrical activation pattern and times. Myocardial strain magnitude and timing are also significantly disrupted. The $E_{cc}$ and $E_{ll}$ strain profiles display reduced peak amplitudes alongside delayed and dispersed peak timing in the LV compared to baseline simulations. The strain curves are temporally prolonged, indicating dispersed mechanical activation and reduced contractility. These results agree with clinical studies that report widened QRS complexes and reduced peak LV pressure in *LBBB* patients [38,39]. The prolonged electromechanical delay in *LBBB* reduces the time available for effective contraction, thereby resulting reduced stroke volume and overall cardiac output [41,58]. In the *LAFB* case, the impact on cardiac function is less severe, with a smaller reduction in the peak LV pressure due to dyssynchronous contraction in the anterior LV wall [59,60]. We note that the *LAFB* case has an SDI value of 5.87%, which is associated with mild LV dysfunction as defined in the clinic [57]. A summary of the key ventricular function indices for all simulated cases is provided in Table 2.

### 4.3. Relationship Between SDI, Activation Time and Cardiac Pump Function

By combining the results from simulations with different degrees and locations of blockage in the Purkinje fiber network, we also establish the relationship between cardiac pump function indices and myocardial activation as well as SDI. The results show that there is a threshold in myocardial activation time where beyond 240 [ms], EF as well as myocardial strains start to decrease significantly. For SDI, a threshold of 8.4% is identified, beyond which the cardiac pump function begins to deteriorate, consistent with findings from clinical studies [57,61]. Ref. [48] showed that SDI values exceeding 5% were associated with significant LV mechanical dyssynchrony and reduced ejection fraction. In contrast, [62] reported that patients with lower global longitudinal strain frequently had SDI above 5%, a combination linked to impaired systolic function and greater potential benefit from cardiac resynchronization therapy (CRT). Several studies propose this SDI threshold as a potential marker for distinguishing between CRT responders and non-responders [63,64]. Elevated SDI values are also employed in pre–CRT evaluation and were associated with significant reductions of LV synchrony and long–term remodeling [57,65]. Collectively, these findings support the use of SDI as a clinically relevant parameter to identify patterns associated with patients who may benefit from CRT, particularly in instances where conventional criteria may be inconclusive. From a clinical perspective, these results highlight how conduction abnormalities within the Purkinje network can reproduce mechanical dyssynchrony seen in patients with bundle branch or fascicular blocks. Such spatiotemporal activation changes are key determinants of CRT success, where both the extent and distribution of electrical activation delays affect ventricular resynchronization and pump function recovery. By linking simulated activation delay, systolic dyssynchrony index, and hemodynamic performance, this framework provides a quantitative link between electrical conduction abnormalities and mechanical outcomes observed clinically. However, the present model is intended to provide mechanistic insight rather than direct clinical prediction or treatment planning. Consequently, the same approach may provide a framework to explore how conduction abnormalities influence CRT or conduction system pacing configurations based on patient-specific conduction remodeling. Clinically, prolonged QRS duration reflects delayed ventricular activation and is closely linked to impaired mechanical synchrony and reduced ejection fraction. Patients with *LBBB* (QRS > 120 [ms]) typically exhibit marked EF reduction and lower strain magnitudes [66,67]. In our simulations, the conduction block cases reproduced similar trends with prolonged activation times corresponding to lower EF and diminishing peak strain, paralleling the clinical QRS–EF relationship.

### 4.4. Comparison with Other Computer Models

This computational study addresses some of the limitations associated with previous studies, which have either focused on electrical activation using simplified conduction approaches or on mechanical performance without modeling the specialized cardiac conduction system. For example, ref. [68] used imaging and motion tracking to create an electromechanical framework but did not include the anatomical structure of the Purkinje fiber system. Other computational studies, such as [69], used personalized cardiac electrophysiological data to predict ablation outcomes in infarct–related tachycardia. These studies, however, focused on electrical propagation and neglected the impact of electrical conduction or blocks on cardiac mechanics. Other more recent work such as [70,71] developed computational models with integrated Purkinje fiber systems to simulate pacing strategies and their effect on cardiac function. These contributions provide key insights into CRT outcomes. Nevertheless, even these advanced models tend to assume static *LBBB* configurations and primarily evaluate lead placement or pacing optimization rather than investigating consequences of the location of the conduction block itself. Our model addresses this gap by incorporating spatially distributed PMJs and simulating variations in conduction blocks, ranging from *LAFB* and *LBBB* of the Purkinje fiber network to cases in remote areas of the Purkinje fiber network (*LSAFB* and *LPFB*). This enables us to quantify different conduction blocks and analyze electrical activation patterns, regional mechanical strains, intraventricular mechanical dyssynchrony and EF. Furthermore, ref. [72] proposed a personalized virtual heart model capable of real–time conduction simulation using a simplified Purkinje fiber network. In contrast, our Purkinje fiber network was verified with experimental data [26] to develop a physiologically grounded framework for the *baseline* but was further used to introduce conduction blocks.

## 5. Limitations

There are several limitations associated with this study. First, the simulation results were obtained based on an idealized left ventricular geometry. While patient–specific LV geometries may improve physiological fidelity, the use of an ellipsoidal geometry was a deliberate modeling choice that allowed us to isolate the mechanistic effects of conduction timing from geometric variability and to ensure numerical robustness during systemic parameter exploration. This simplification allowed us a clearer interpretation of the impact of Purkinje fiber network abnormalities on the global LV pump function. The modular computational framework allows versatility and can incorporate patient–specific LV geometry in the future.

Second, a single fractal tree Purkinje fiber network was utilized rather than a variety of subject–specific anatomical reconstructions, which is a common strategy in computational electromechanics modeling. Although the resulting myocardial activation maps demonstrate distributed endocardial breakthrough and transmural propagation, the precise location and timing of early activation depend on the generated network topology. Anatomical and stochastic variability in branching structure were not explicitly considered in this study. Future analysis of the Purkinje fiber network structure will enhance physiological fidelity.

Third, we do not consider the impact of the right ventricle and the pulmonary circulation and correspondingly, we do not account for interventricular mechanical interactions or interseptal dyssynchrony. The present formulation also omits possible retrograde conduction from the right Purkinje bundle branch or through the myocardium into the left Purkinje bundle branch, which has been observed clinically and may influence activation timing near the septum [73]. In clinical LBBB, transseptal activation and residual Purkinje conduction from the RV may partially shorten the total activation. In the absence of these mechanisms, the present LV framework may therefore result in prolonged global QRS durations compared to clinical observations. Incorporating a bi–ventricular geometry with bidirectional Purkinje–myocardial coupling and interventricular septal mechanics would provide a more complete representation of ventricular coordination.

Fourth, we employed a phenomenological electrophysiology model for both the LV and the Purkinje fiber network based on the modified FitzHugh–Nagumo formulation [28]. This model reproduced threshold–dependent excitation and recovery with low computational cost, allowing efficient coupling to mechanics and circulation. However, the FHN formulation does not explicitly represent ionic currents, calcium transients, or restitution behavior and thus may underestimate local dispersion of activation and rate–dependent conduction variability. Moreover, activation transfer across the Purkinje–myocardial junctions (PMJs) was modeled using a threshold–based rule, where a myocardial stimulus is triggered once the Purkinje potential exceeds a predefined threshold. While this approach ensures robust and numerically stable coupling between the Purkinje and myocardial domains, it does not capture graded current exchange or retrograde conduction from myocardium to Purkinje fibers. These simplifications may affect local activation timing but are not expected to alter the global mechanical trends reported here, which primarily depend on overall activation delay rather than detailed ionic dynamics.

Fifth, the present model focuses on anterograde activation under sinus rhythm conditions and does not account for retrograde conduction or reentrant electrophysiological mechanisms. These phenomena may contribute to arrhythmogenesis in conditions such as bundle branch block and are therefore beyond the scope of the current study.

Last, we employed a Purkinje fiber network that was generated using a hybrid deterministic–stochastic algorithm, which may not capture all the details of the network. Nevertheless, we ensured that the distribution of PMJs in the Purkinje fiber network is consistent with general measurements. Overall, these simplifications enable a stable and computationally efficient framework for mechanistic analysis, while future bi–ventricular and electrophysiological refinements will improve physiological fidelity of the established computational model.

## 6. Conclusions

We developed a computational modeling framework that couples cardiac conduction in the Purkinje fiber network with LV electromechanics, enabling systematic investigation of conduction abnormalities and the effect on cardiac function. Using the computational framework, we investigated the effects of different conduction blocks occurring in the Purkinje fiber network on LV pump function and showed that there exists a threshold in myocardial activation time (240 [ms]) and SDI (8.4%) beyond which EF and myocardial strains start to decrease exponentially. These findings provide a mechanistic basis that may be useful for assessing the impact of conduction abnormalities using clinically measured indices on cardiac pump function. The modeling framework also serves as a foundation for the development of future patient–specific simulations to guide cardiac resynchronization therapy planning and optimization.

## Figures and Tables

**Table 1:** Normalized systolic dyssynchrony index (SDI) values computed from circumferential strain ($SDI_{E_{cc},norm}$) for *baseline*, *LAFB*, *LSAFB*, *LPFB*, and *LBBB* activation scenarios.

	${\mathrm{SDI}}_{E_{\mathrm{cc}},\mathrm{norm}}$ [%]
*Baseline*	1.5026
*LAFB*	5.8664
*LSAFB*	1.4255
*LPFB*	1.6494
*LBBB*	14.5357

**Table 2:** Quantitative comparison of simulated left–ventricular functional indices with literature values. Comparison of ejection fraction (EF), end–diastolic volume (EDV), end–systolic volume (ESV), peak left–ventricular pressure (LVP), and systolic dyssynchrony index (SDI) obtained from the present baseline simulation and representative values reported in previous experimental and computational studies.

	EF [%]	EDV [mL]	ESV [mL]	Peak LVP [mmHg]	${\mathrm{SDI}}_{E_{\mathrm{cc}},\mathrm{norm}}$ [%]
*Baseline*	59	89.7	36.6	123	1.5026
Anterior block (*LAFB*)	58	89.7	37.2	116	5.8664
Septal-apical block (*LSAFB*)	59	89.7	36.0	122	1.4255
Posterior block (*LPFB*)	59	89.7	36.0	122	1.6494
Total block (*LBBB*)	46	84.0	45.3	96	14.5357
Reported range/ literature values	52–73 (normal) [46]	56–104 (women);	19–49 (women);	90–140 (normal) [47]	4.1 ± 2.2 (normal) [48]
	35–50 (LBBB with HF) [49]	67–155 (men) [50]	22–58 (men) [50]		13.4 ± 8.1 (HF) [48]

**Figure A1:**
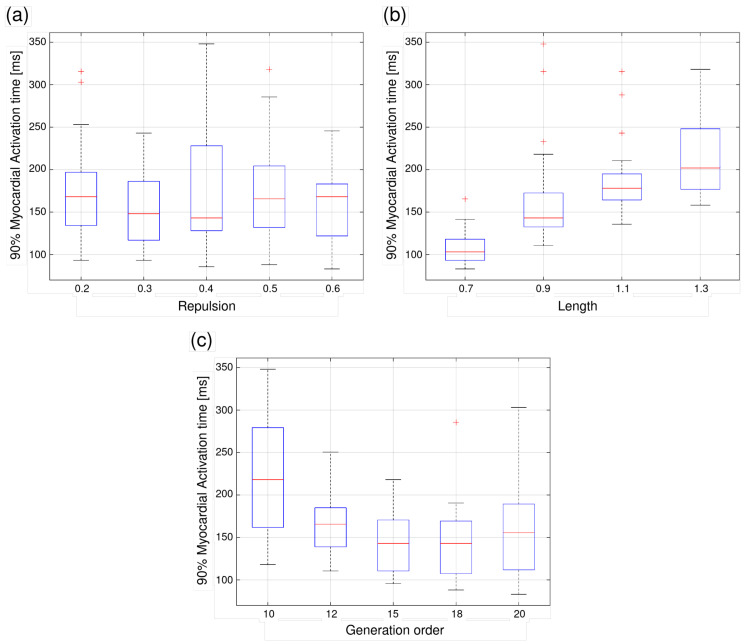
Sensitivity of 90% myocardial activation time to variations in Purkinje fiber network generation parameters: (**a**) *Repulsion*, (**b**) *Length*, and (**c**) *Generation order*.

## Data Availability

The original data for this study is included in the article/Appendix A and are openly available in Effects of Purkinje Fiber Conduction Block on Cardiac Pump Function at: https://github.com/hagersan/Effects-of-Purkinje-Fiber-Conduction-Block-on-Cardiac-Pump-Function (accessed on 13 April 2025).

## Appendix A

An implicit time integration scheme was employed for temporal discretization of the monodomain equation and the resultant weak form for time step n + 1, which is given as:

(A1) $$\int_{\Omega_{0}}\left(\phi -\phi_{n}\right)\;\Delta t^{-1}\delta \phi =-\int_{\Omega_{0}}D\nabla \phi \cdot \nabla \;\delta \phi +\int_{\Omega_{0}}\left(f_{\phi }+I_{s}\right)\;\delta \phi ,$$

(A2) $$\int_{\Omega_{0}}\left(r-r_{n}\right)\;\Delta t^{-1}\;\delta r=\int_{\Omega_{0}}g_{r}\;\delta r,$$

where Δt is the time step, $\phi_{n}$ and $r_{n}$ are the values from the previous time step, D is the diffusion tensor, $f_{\phi }$ and $f_{r}$ are the reaction terms, and $I_{s}$ denotes the external stimulus current. For the mechanical equilibrium equations, a stabilized linear interpolation scheme was employed to discretize both the displacement field u and pressure field *p*. At each time step, the following weak form of the pressure–driven mechanical equilibrium equation

(A3) $$\begin{array}{cc} L\left(u,p\right)= & \int_{\Omega_{0}}\left(\mathrm{FS}-pF^{-T}\right):\nabla \;\delta u\;dV\;-\int_{\Omega_{0}}\delta p\;\left(J-1\right)\;dV\;\\ \; & +\int_{\Gamma_{\mathrm{epi}}}\left(K_{epi}\;u+C_{epi}\;\dot{u}\right)\cdot \delta u\;dS-P_{LV}\int_{\Gamma_{\mathrm{endo}}^{LV}}JF^{-T}N\cdot \delta u\;dS\\ \; & -\sum_{n_{el}}\frac{\alpha h^{2}}{2\mu }\int_{\Omega_{e}}\nabla p\cdot \nabla \delta p\;dV \end{array}$$

was solved. The last term in Equation (A3) is an element–wise stabilization term to avoid volumetric locking and pressure instabilities associated with nearly incompressible materials and weakly enforcing incompressibility [74]. In that term, α represents a stabilization parameter, *h* is the element mesh size and μ is the local shear modulus. In this formulation (Equation (A3)), a Robin–type boundary condition was imposed on the epicardial surface as:

(A4) $$\mathrm{PN}+K_{epi}\;u+C_{epi}\;\dot{u}=0,$$

where $P=\mathrm{FS}-pF^{-T}$, represents the first Piola–Kirchhoff stress tensor, N is the outward normal with respect to the epicardial surface, u is the displacement field and $K_{epi}$ denotes the stiffness tensor with a normal ($k_{⊥}$) and a tangential ($k_{\|}$) directional component, e.g.:

(A5) $$K_{epi}=k_{⊥}\left(N⊗N\right)+k_{\|}\left(I-N⊗N\right).$$

Similarly, $C_{epi}$ is the designated damping tensor split into normal and tangential directions $c_{⊥}$ and $c_{\|}$, i.e.,

(A6) $$C_{epi}=c_{⊥}\left(N⊗N\right)+c_{\|}\left(I-N⊗N\right).$$

The contribution of $K_{epi}$ and $C_{epi}$ ensures physiological tangential mobility while constraining against radial expansion of the heart, resembling the in vivo pericardial constraint imposed on the heart [75]. In this formulation, left ventricular pressure $P_{LV}$ was also imposed on the endocardial surface as a Neumann boundary condition, circumventing the artificial pressure build up often observed in volume–driven approaches. The resulting nonlinear system of equations was solved monolithically using a Newton–Raphson method with consistent tangent operators.

Table A1 contains the input parameters for the Purkinje fiber network generation algorithm, including those defining the trunk of the network. Subsequent branch generations are categorized into mid–network region, representing the overall geometry of the Purkinje fiber network and a terminal region associated with the number of terminal nodes.

**Table A1:** Summary of the driving parameter set of the Purkinje fiber network, including trunk length, bifurcation angles, generation order for the individual segments, and number of terminal nodes (associated with PMJs).

Trunk	*Initial node*	[−0.57, −1.88, −0.168]	[cm]
	*Initial direction*	[0.0, 0.0, −1.0]	[-]
	*Initial length*	1.5	[cm]
	*Generation order*	0	[-]
	*Branch angle*	1.8	[rad]
	*Length*	1.8	[cm]
	*Segment length*	0.18	[cm]
	*Fascicles length*	0.6	[cm]
	*Fascicles angle*	0.7	[rad]
Mid area network	*Generation order*	15	[-]
	*Branch angle*	0.15	[rad]
	*Length*	0.5	[cm]
	*Segment length*	0.1	[cm]
	*Fascicles length*	1.5	[cm]
	*Fascicles angle*	[−1.5, 0.2]	[rad]
	*Repulsion*	0.3	[-]
Terminal area network	*Generation order*	8	[-]
	*Branch angle*	0.15	[rad]
	*Length*	0.8	[cm]
	*Segment length*	0.4	[cm]
	*Fascicles length*	0.5	[cm]
	*Fascicles angle*	[−1.5, 0.2]	[rad]
	*Repulsion*	0.3	[-]
Network	Number of terminal nodes (PMJs)	582	[-]

Table A2 contains the input parameters for the Purkinje and myocardial electrophysiological models, discussed in Equation (1) for the baseline case of the conducted simulations.

**Table A2:** Baseline parameter set for electrophysiological model, where $I_{stim_{pj}}$ denotes the initial stimulus current for the Purkinje fiber network, $I_{stim_{myo}}$ represents the stimulus transferred from the terminal nodes of the Purkinje fibers to the ventricular myocardium, $d_{pj}$ is the electrical conductivity within the Purkinje network in Siemens per meter [S/m], $d_{iso_{myo}}$ refers to the electrical conductivity within the extracellular matrix of the myocardium in [S/m], and $d_{ani_{myo}}$ is the electrical conductivity in [S/m] along the cardiomyocyte fiber direction.

Variable		Unit
$I_{stim_{pj}}$	25.0	[-]
$I_{stim_{myo}}$	1.0	[-]
$d_{pj}$	150	[S/m]
$d_{iso_{myo}}$	0.01	[S/m]
$d_{ani_{myo}}$	0.2	[S/m]

Table A3 summarizes the input parameters for the Purkinje and myocardial electrophysiological models, discussed in Equation (1) for the localized blocks in the Purkinje network of the conducted simulations.

**Table A3:** Parameter set for electrophysiological model with localized conduction blocks in the Purkinje network, where $I_{stim_{pj}}$ denotes the initial stimulus for the Purkinje fiber network, $I_{stim_{myo}}$ represents the stimulus transferred from the terminal nodes of the Purkinje fibers to the ventricular myocardium, $d_{pj}$ is the electrical conductivity within the Purkinje network in [S/m], $d_{iso_{myo}}$ refers to the electrical conductivity within the extracellular matrix of the myocardium in [S/m], and $d_{ani_{myo}}$ is the electrical conductivity along the cardiomyocyte fiber direction in [S/m].

Variable		Unit
$I_{stim_{pj}}$	25.0	[-]
$I_{stim_{myo}}$	1.0	[-]
$d_{pj}$	150	[S/m]
$d_{iso_{myo}}$	0.001	[S/m]
$d_{ani_{myo}}$	0.01	[S/m]

Table A4 and Table A5 summarize the parameters for the trend line fitting of the relationship between dyssynchrony and global cardiac function with a stretched exponential function and an explicit plateau phase at the start with respect to 90% myocardial activation and $SDI_{E_{cc},norm}$, respectively, i.e.,

(A7) $$y\left(x\right)=c+Ae^{\left(-k{\left|x-x_{0}\right|}^{p}\right)}.$$

Here, $x_{0}$ indicates the transition point of the data before the decrease, while c+A is the plateau of the data. The exponent p>1 controls the duration of the plateau and *k* defines the steepness of the drop. The trend line was defined via a low-parameter model and only optimizes *k* while solving for *A* and *c* by simple least squares for each *k*, whereas *p* and $x_{0}$ are fixed.

**Table A4:** Parameter set of the stretched-exponential trendline fitted to EF, $E_{cc}$ and $E_{ll}$ against 90% myocardial activation time [ms], where *A* is the amplitude (plateau minus asymptote), *k* [$ms^{-p}$] controls the decline rate, *c* is the offset for c+A as plateau level, $x_{0}$ [ms] is the transition point before the drop, p>1 is the shape exponent, and $R^{2}$ represents the coefficient of determination.

	*A*	*k*	*c*	$x_{0}$	*p*	$R^{2}$
EF	0.16	0.13 $\times \;10^{-11}$	0.42	−6.56	4.2	0.98
$E_{cc}$	0.07	0.17 $\times \;10^{-8}$	0.16	25.94	3	0.99
$E_{ll}$	0.05	1.43 $\times \;10^{-10}$	0.14	30	3.8	0.98

**Table A5:** Parameter set of the stretched-exponential trendline fitted to EF, $E_{cc}$ and $E_{ll}$ against $SDI_{E_{cc},norm}$ [-], where *A* is the amplitude (plateau minus asymptote), *k* [$ms^{-p}$] controls the decline rate, *c* is the offset for c+A as plateau level, $x_{0}$ [ms] is the transition point before the drop, p>1 is the shape exponent, and $R^{2}$ represents the coefficient of determination.

	*A*	*k*	*c*	$x_{0}$	*p*	$R^{2}$
EF	0.19	1.99 $\times \;10^{-5}$	0.39	1.43	4.2	0.98
$E_{cc}$	0.07	5.59 $\times \;10^{-5}$	0.16	1.43	3.8	0.97
$E_{ll}$	0.05	1.99 $\times \;10^{-5}$	0.14	1.43	4.2	0.95

Table A6 summarizes the variations in electrophysiological input parameters used in the sensitivity analysis described in Section 3.3. These parameter perturbations were introduced to assess the influence of myocardial and Purkinje fiber network conductivity on activation patterns in the *baseline*, *LAFB*, incomplete *LAFB*, *LSAFB*, incomplete *LSAFB*, *LPFB*, incomplete *LPFB*, and *LBBB* simulation cases.

**Table A6:** Perturbation of myocardial input parameters for the sensitivity analysis conducted in Section 3.3.

	Color	$I_{{\mathrm{stim}}_{\mathrm{myo}}}$ [-]	$d_{{\mathrm{iso}}_{\mathrm{myo}}}$ [S/m]	$d_{{\mathrm{ani}}_{\mathrm{myo}}}$ [S/m]	$d_{\mathrm{pj}}$ [S/m]
*Baseline*	•	1.0	0.02	0.4	150
	•	1.0	0.005	0.05	150
	•	1.0	0.008	0.15	150
	•	1.0	0.01	0.2	150
	•	1.0	0.008	0.18	150
Anterior block (*LAFB*)	•	0.4	0.02	0.15	150
	•	0.3	0.02	0.15	150
	•	0.28	0.02	0.15	150
	•	0.26	0.02	0.15	150
	•	0.26	0.002	0.015	150
	•	0.24	0.002	0.015	150
	•	0.25	0.001	0.01	150
	•	0.25	0.005	0.05	150
	•	0.25	0.01	0.07	150
	•	0.24	0.01	0.07	100
	•	0.5	0.02	1.5	150
	•	0.5	0.002	0.5	150
	•	0.5	0.0002	0.05	150
	•	0.5	0.00002	0.005	150
	•	0.5	8 $\times \;10^{-6}$	0.002	150
	•	0.5	8 $\times \;10^{-6}$	0.002	150
	•	1.0	0.001	0.01	150
	•	1.0	0.0005	0.005	150
	•	1.0	0.01	0.2	150
	•	1.0	0.005	0.05	150
	•	1.0	0.0006	0.006	150
Incomplete anterior block—with delay (*iLAFB*)	•	1.0	0.001	0.01	150
Septal-apical block (*LSAFB*)	•	1.0	0.001	0.01	150
	•	1.0	0.0005	0.005	150
Incomplete septal-apical block—with delay (*iLSAFB*)	•	1.0	0.001	0.01	150
Posterior block (*LPFB*)	•	1.0	0.001	0.01	150
	•	1.0	0.0005	0.005	150
Incomplete posterior block—with delay (*iLPFB*)	•	1.0	0.001	0.01	150
Total block (*LBBB*)	•	1.0	0.001	0.01	150
	•	1.0	0.003	0.03	150
	•	1.0	0.001	0.01	100
	•	0.5	0.001	0.01	150
	•	0.9	0.003	0.03	150
	•	0.8	0.003	0.03	150
	•	0.79	0.003	0.03	150
	•	0.75	0.003	0.03	150

To quantify the influence of the generated Purkinje fiber network geometry on activation dynamics, a comprehensive parameter sweep was performed across geometrical parameter combinations of *Repulsion*, *Generation order*, and *Length*. The resulting network characteristics and activation metrics for all geometries are summarized in Table A7, while the corresponding statistical relationships are evaluated through correlation analysis in Table A8. The analysis shows that geometric parameters influence the number of terminal nodes, with *Generation order* exhibiting a moderate positive correlation (≈0.43) and *Length* a strong negative correlation (≈−0.74), while *Repulsion* has only a minor effect. Furthermore, 90% myocardial activation time demonstrates a strong inverse relationship with the number of terminal nodes (≈−0.78), indicating that terminal nodes play a dominant role in governing activation dynamics. These results suggest that geometric parameters affect activation primarily indirectly through their influence on Purkinje fiber network structure and PMJ distribution. Additional sensitivity trends for individual parameters are shown in Figure A1.

**Table A7:** Sensitivity analysis of Purkinje fiber network generation parameters. This table summarizes the various combinations of generation parameters for Purkinje fiber networks with the focus on *Repulsion* (*r*), *Generation order* ($N_{mid}$) and *Length* ($len_{mid}$) in the mid-network region, resulting in a certain number of terminal nodes (Terminalnodes), 90% Purkinje fiber network activation time, and 90% myocardial activation time in milliseconds. A value of −1 for the 90% activation time indicates that no activation was observed within the simulated time window.

*Repulsion*	$N_{\mathrm{mid}}$	${\mathrm{len}}_{\mathrm{mid}}$	*Terminal Nodes*	90% PJ Activation Time [ms]	90% Myocardial Activation Time [ms]
0.2	10	0.7	126	20.5	125.5
0.2	10	0.9	71	20.5	315.5
0.2	10	1.1	77	20.5	315.5
0.2	10	1.3	70	23	253
0.2	12	0.7	186	20.5	110.5
0.2	12	0.9	96	20.5	185.5
0.2	12	1.1	112	23	200.5
0.2	12	1.3	85	23	170.5
0.2	15	0.7	216	23	115.5
0.2	15	0.9	135	23	193
0.2	15	1.1	97	23	193
0.2	15	1.3	85	20.5	158
0.2	18	0.7	281	23	93
0.2	18	0.9	154	25.5	143
0.2	18	1.1	116	23	150.5
0.2	18	1.3	76	23	165.5
0.2	20	0.7	273	23	95.5
0.2	20	0.9	151	25.5	150.5
0.2	20	1.1	105	23	173
0.2	20	1.3	65	20.5	303
0.3	10	0.7	124	20.5	118
0.3	10	0.9	111	23	160.5
0.3	10	1.1	83	23	243
0.3	10	1.3	60	23	−1
0.3	12	0.7	165	23	113
0.3	12	0.9	81	23	233
0.3	12	1.1	96	23	148
0.3	12	1.3	55	25.5	−1
0.3	15	0.7	237	25.5	95.5
0.3	15	0.9	148	23	138
0.3	15	1.1	105	23	148
0.3	15	1.3	59	25.5	−1
0.3	18	0.7	236	25.5	103
0.3	18	0.9	156	25.5	120.5
0.3	18	1.1	100	23	185.5
0.3	18	1.3	79	23	188
0.3	20	0.7	286	25.5	93
0.3	20	0.9	164	23	140.5
0.3	20	1.1	110	25.5	180.5
0.3	20	1.3	90	25.5	203
0.4	10	0.7	129	20.5	128
0.4	10	0.9	52	20.5	348
0.4	10	1.1	56	20.5	288
0.4	10	1.3	68	23	228
0.4	12	0.7	171	23	118
0.4	12	0.9	119	25.5	138
0.4	12	1.1	107	23	165.5
0.4	12	1.3	79	23	250.5
0.4	15	0.7	272	23	98
0.4	15	0.9	155	25.5	133
0.4	15	1.1	123	23	155.5
0.4	15	1.3	64	25.5	−1
0.4	18	0.7	273	25.5	103
0.4	18	0.9	173	25.5	148
0.4	18	1.1	109	25.5	135.5
0.4	18	1.3	67	25.5	−1
0.4	20	0.7	288	25.5	85.5
0.4	20	0.9	173	25.5	128
0.4	20	1.1	116	25.5	203
0.4	20	1.3	84	25.5	240.5
0.5	10	0.7	76	20.5	165.5
0.5	10	0.9	83	20.5	218
0.5	10	1.1	73	23	210.5
0.5	10	1.3	71	23	318
0.5	12	0.7	129	23	141.5
0.5	12	0.9	145	25.5	145.5
0.5	12	1.1	120	23	165.5
0.5	12	1.3	82	23	183
0.5	15	0.7	235	25.5	105.5
0.5	15	0.9	175	25.5	125.5
0.5	15	1.1	120	23	168
0.5	15	1.3	90	23	218
0.5	18	0.7	281	23	88
0.5	18	0.9	177	23	133
0.5	18	1.1	130	23	160.5
0.5	18	1.3	84	23	285.5
0.5	20	0.7	295	25.5	90.5
0.5	20	0.9	179	23	130.5
0.5	20	1.1	116	23	178
0.5	20	1.3	90	25.5	198
0.6	10	0.7	133	20.5	130.5
0.6	10	0.9	97	20.5	168
0.6	10	1.1	79	20.5	193
0.6	10	1.3	86	20.5	245.5
0.6	12	0.7	148	20.5	115.5
0.6	12	0.9	109	23	168
0.6	12	1.1	115	23	178
0.6	12	1.3	92	20.5	200.5
0.6	15	0.7	284	23	95.5
0.6	15	0.9	194	23	110.5
0.6	15	1.1	117	25.5	188
0.6	15	1.3	81	23	170.5
0.6	18	0.7	286	23	90.5
0.6	18	0.9	177	25.5	135.5
0.6	18	1.1	135	25.5	170.5
0.6	18	1.3	98	23	190.5
0.6	20	0.7	302	23	83
0.6	20	0.9	178	25.5	128
0.6	20	1.1	131	23	173
0.6	20	1.3	85	23	160.5

**Table A8:** Correlation analysis between Purkinje fiber network parameters, terminal nodes, and activation times across different Purkinje fiber network geometries. Myocardial activation time shows a strong inverse relationship with the number of terminal nodes, indicating that terminal nodes are a driving force for activation dynamics. All reported values correspond to dimensionless correlation coefficients.

Parameter	Correlation with Terminal Nodes	Correlation with 90% Myocardial Activation Time	Correlation with 90% Purkinje Fiber Network Activation Time
*Repulsion*	0.0734	−0.0896	0.0404
*Generation order*	0.4291	−0.3415	0.5976
*Length*	−0.7364	0.6564	−0.0415
Terminal nodes	–	−0.7775	0.3780
